# Synthesis and Characterization of a Superabsorbent Polymer Gel Using a Simultaneous Irradiation Technique on Corn Straw

**DOI:** 10.3390/gels11040244

**Published:** 2025-03-26

**Authors:** Xingkui Tao, Jun Guo, Aihua Wang, Qiang Wang, Yang Yang, Minwei Xu

**Affiliations:** 1 College of Biology and Food Science, Suzhou University, Suzhou 234000, China; 2 Department of Plant Sciences, North Dakota State University, Fargo, ND 58108, USA

**Keywords:** irradiation, corn straw, graft, water absorption, superabsorbent gels

## Abstract

Utilizing gamma rays as an initiating agent, a simultaneous irradiation method was applied to graft acrylic acid and acrylamide onto corn straw that had been decrystallized using a NaOH/urea solution at a reduced temperature, aiming to fabricate superabsorbent polymer gel (SAPG) capable of absorbing significantly more water. The structural attributes of the corn straw, the decrystallized corn straw, and the SAPG were analyzed via Fourier transform infrared spectroscopy (FTIR), X-ray crystal powder diffraction (XRD), thermogravimetric analysis (TG), and scanning electron microscopy (SEM). To enhance the SAPG’s performance, optimization of various parameters was carried out, such as irradiation dose, dose rate, the ratio of monomer to corn straw, the proportion of acrylic acid (AA) to acrylamide (Am), and the degree of neutralization. The resulting SAPG exhibited distilled water absorption of 1033 g/g and 90 g/g in 0.9 wt.% NaCl solution, with a radiation dose of 5 kGy, a dose rate of 1.5 kGy/h, AA-to-AM mass ratio of 1.2, monomer-to-CS mass ratio of 7, and 90% AA neutralization.

## 1. Introduction

Superabsorbent polymer gels (SAPGs) are distinctive materials known for their crosslinked chains, which create a three-dimensional network [1,2]. These chains are embedded with numerous hydrophilic groups, enabling SAPGs to soak up vast quantities of water—exceeding a thousandfold of their initial weight—and become hydrogels [3]. The hydrogels’ equilibrium swelling enables them to hold onto considerable amounts of aqueous fluid, even when under pressure [4]. SAPGs are widely used in various fields, including personal care, agriculture, horticulture, drug delivery systems, and coal dewatering [5]. SAPGs can be divided into two varieties: those synthesized using oil-based chemicals like AA, Am, and acrylonitrile and those synthesized through graft copolymerization [6]. Synthetic gels are less biodegradable and utilize natural resources, negatively impacting the environment. Conversely, grafted SAPGs, derived from biodegradable and eco-friendly biopolymers, have garnered significant attention due to their abundant, non-toxic, and cost-effective resources, such as AA, Am, and grafting to corn straw [3].

Grafting SAPGs typically employs polysaccharides such as starch, cellulose, chitosan, and other bio-resources [5,7,8,9]. Cellulose, a primary constituent of crop residues, is a plentiful biopolymer consisting of C_6_H_10_O_5_ units linked by β-1,4-glucosidic bonds. Each unit has three hydroxyl groups that create a robust hydrogen bond network, resulting in a crystalline 3D biopolymer structure which makes cellulose insoluble in water, even at elevated temperatures [10,11,12]. Unlike starch, cellulose does not gelatinize, preventing monomer molecules from interacting with the cellulose interior during grafting and reducing grafting efficiency [13,14]. A previous work created SAPGs by grafting cellulose with hydrophilic monomers without disrupting the crystalline structure, thus limiting the gels’ performance when using cellulose and vinyl monomers under heterogeneous conditions [15].

To enhance grafting efficiency, biopolymers must be unfolded before grafting, with gelatinized starch being an example of this. Similarly, the decrystallization of the natural cellulose in straw is required for SAPG production. Studies have shown that it is possible to produce SAPGs by grafting cellulose and monomers under homogeneous conditions, such as through alkali-freezing treatments that break crystalline linkages, facilitating cellulose decrystallization in a cold NaOH/urea solution [16,17].

According to the research conducted by Liu et al. [18], AA can be grafted onto the side chains of wheat straw cellulose without decrystallization to form SAPGs with absorbencies of 417 g/g in distilled water and 45 g/g in 0.9 wt% NaCl solution. Benamer et al. used gamma rays of ^60^Co as an initiator for the synthesis of chitosan beads grafted with AA for metal ion sorption [19]. The gamma rays of ^60^Co as an initiator can overcome the disadvantages of high pollution and high energy consumption of chemical catalysts.

However, based on the current literature, few studies have utilized radiation technology to graft AA and Am to cellulose from corn straw to synthesize SAPGs. Therefore, this research attempted to graft AA and Am onto the cellulose of corn straw using gamma rays of ^60^Co as the initiators.

In order to rationally utilize the resources of corn straw, a simple and low-cost SAPG based on AA and Am with N,N-methyl-enebisacrylamide (MBA) as an initiator and crosslinker was synthesized [20]. The process involved dissolving corn straw in a NaOH/urea aqueous solution to achieve decrystallized cellulose, with AA neutralizing the solution to prevent Am decomposition in the alkaline medium. An SAPG was produced through simultaneous irradiation using gamma rays as the initiators under various conditions. The absorption characteristics of the SAPG were then correlated with its structural, compositional, and environmental factors, as well as the solution’s nature. This study introduced a novel method of using crop residue for SAPG production, potentially reducing production costs, enhancing environmental friendliness.

## 2. Results and Discussion

### 2.1. Analysis of the Structure and Morphology of the Sample

#### 2.1.1. XRD Analysis

**Figure 1:**
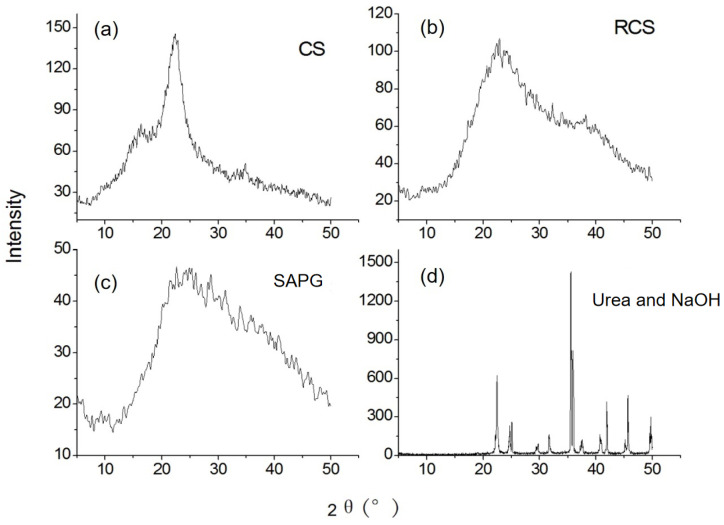
X-ray diffraction (XRD) patterns: (**a**) corn straw (CS), (**b**) regenerated corn straw (RCS), (**c**) superabsorbent polymers gel (SAPG), and (**d**) mixture of urea and NaOH.

The XRD patterns for CS, RCS, SAPG, and urea are depicted in Figure 1. The raw CS (Figure 1a) exhibits two distinct diffraction peaks, characteristic of its polycrystalline (cellulose) structure. Following the decrystallization process using the NaOH/urea-freezing aqueous solution, these peaks largely disappeared and converted into a broad, extensive dispersion peak. This suggests the successful disruption of crystalline linkages within the CS cellulose sheets. The resultant destruction of the hydrogen-bonded crystal structure between the hydroxyl groups of the β-1,4-glucosidic bond chains in CS results in the transformation of the compact polycrystalline structure into a loose, amorphous one. Figure 1b shows the RCS. Consequently, low-molecular-weight monomers (AA or Am) can readily penetrate the cellulose matrix, extensively contacting the carbohydrate chains. The SAPG’s XRD pattern in Figure 1c reveals poor symmetry and tacticity in the graft copolymer, further transitioning into an amorphous state. The NaOH in the product did not form crystals. Figure 1d shows that the urea XRD pattern demonstrated clear diffraction peaks, signifying its highly crystalline nature. SAPG did not exhibit diffraction peaks, indicating the uniform distribution of urea within the gels [3,11].

#### 2.1.2. IR Analysis

**Figure 2:**
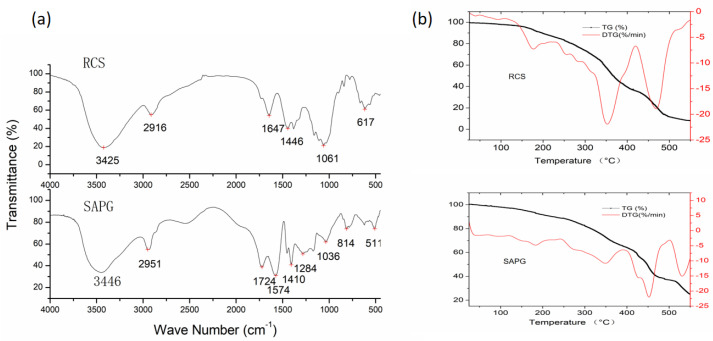
(**a**) Infrared spectra of RCS and SAPG; (**b**) TG and DTG of RCS and SAPG.

The IR spectra of the RCS and SAPG presented in Figure 2a show that RCS primarily consists of cellulose, hemicellulose, and lignin. The raw RCS IR spectrum shows the following bands: the band at 3425 cm^−1^ corresponds to OH stretching in hydroxyl functional groups; the band at 2916 cm^−1^ is assigned to C-H stretching and bending absorption bands, likely present in the methylene groups of cellulose; the bands at 1647 and 1446 cm^−1^ are attributed to C=C stretching vibrations in aromatic rings; and the band at 1061 cm^−1^ corresponds to C-O stretching vibrations. The SAPG IR spectrum displays distinct absorption bands. The band at 2528 cm^−1^ is ascribed to N-H stretching, and the bands at 1724 and 1574 cm^−1^ are ascribed to the amide group’s characteristic absorption, possibly including urea’s amide group absorption. The band at 1420 cm^−1^ represents the symmetric stretching vibration of C=O in COOH groups, and the band at 1036 cm^−1^ is attributed to the C-O stretching vibration of -COOH groups. The bands at 1284 cm^−1^ and 814 cm^−1^ are assigned to the planar rocking vibration of C-H of long carbon chains and C=O bending vibrations, respectively, indicating the grafting of acrylic acid and acrylamide onto the RCS cellulose chain [21,22].

#### 2.1.3. Thermal Stability

The TGA and DTG curves for RCS and SAPG are shown in Figure 2b, and the RCS TG curve aligns with findings in the previous literature. RCS degradation occurs in three stages [3]. The initial stage, between 25 °C and 200 °C, involves approximately 2% weight loss, potentially due to water desorption. The second stage, between 200 °C and 300 °C, shows rapid decomposition, with about 60% weight loss, attributed to the dehydration of saccharide rings and the breakage of C-O-C bonds in the RCS cellulose structure. Water molecules may form between neighboring carboxylic or hydroxyl groups of the polymer chains due to anhydride formation and main-chain scission. The third stage, between 300 °C and 550 °C, involves the formation of C=O from the polymeric backbone’s C and O. The SAPG also exhibits three degradation stages. The first, between 25 °C and 200 °C, involves approximately 2% weight loss. There is no rapid decomposition between 200 °C and 300 °C, indicating the higher thermal stability of gels compared to RCS. AA or Am molecules may graft onto RCS, with the chemical bonds between RCS and polymeric (AA-Am) chains enhancing SAPG stability. In summary, SAPG possesses superior thermal stability compared to RCS [15].

#### 2.1.4. SEM Analysis

**Figure 3:**
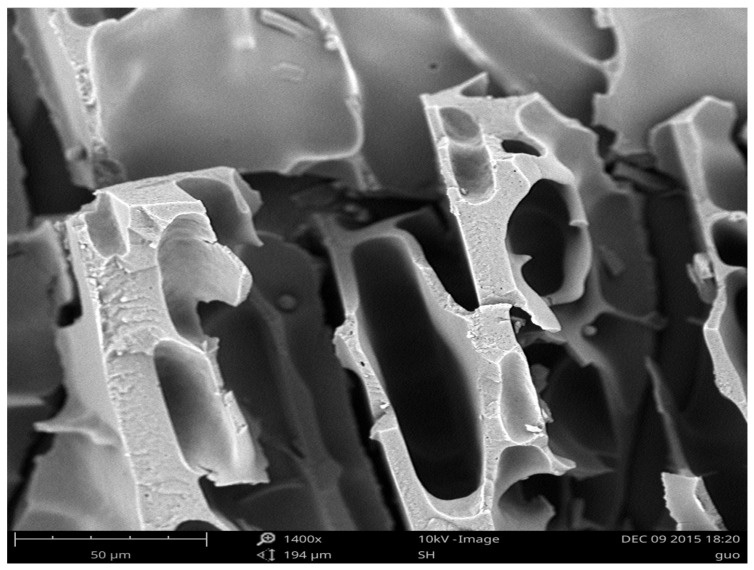
SEM of SAPG.

In Figure 3, we can see that, after polymerization, more porous structures appear on the surface and indeed aided in the absorption of more liquid material. The corn straw exhibits a multilayered structure with impacted characteristics, while RCS possesses a compact and smooth surface. Following the NaOH/urea decrystallization process, the morphology transforms into a loose and rough texture featuring a wavy surface structure. This transformation facilitates the penetration of reagents into the molecular chain, thereby enhancing the rate of chemical modification. Post polymerization, a more porous surface structure emerges, which is beneficial for the absorption of additional liquid material [8,23].

### 2.2. Single-Factor Experiment

#### 2.2.1. Effect of Monomer-to-RCS Ratio on the Water Absorbency of SAPG

**Figure 4:**
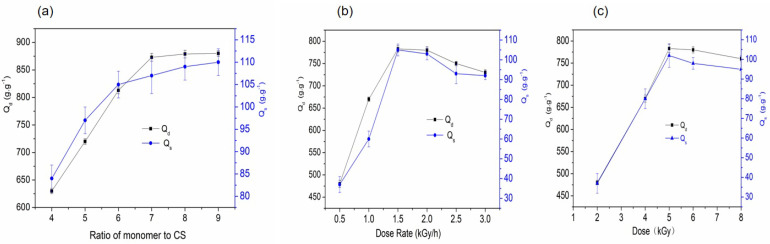
(**a**) Effect of monomer-to-RCS ratio on the water absorption rate. (**b**) Effect of radiation dose rate on the water absorption rate. (**c**) Effect of radiation dose on the water absorption rate.

The addition of crosslinking agents was 1.2% (m/V) at a dose of 4.5 kGy, dose rate of 2.0 kGy/h, AA neutralization of 80%, and m(AA):m(Am) of 2 [15]. The impact of the monomer-to-RCS weight ratio, ranging from 4:1 to 9:1, on the absorption rates is presented in Figure 4a. SAPG absorption increased proportionally with the monomers, reaching maximum values at a ratio of 8:1 in distilled water (873 g/g) and 9:1 in the 0.9 wt.% NaCl solution (87 g/g). An increased monomer concentration may boost crosslinking density and promote a three-dimensional network structure in the polymer [19]. Higher monomer concentrations led to a higher grafting ratio, with numerous AA and Am molecules grafted onto the RCS backbone, forming a hydrophilic structure capable of absorbing substantial amounts of aqueous substances. Excessive AA and AM may result in more homopolymers, shielding the RCS radical and impeding further grafting onto cellulose, hemicellulose, and lignin. This facilitates chain transfer reactions, increasing branching reactions and enhancing the polymer’s ability to absorb and retain high amounts of aqueous substances, forming a stiff and rigid structure [24]. Considering the desire for more environmentally friendly products containing increased biological materials, a monomer-to-RCS ratio of 8:1 was utilized.

#### 2.2.2. Effect of Dose Rate on the Water Absorbency of SAPG

As the monomer-to-RCS weight ratio was 8, the dose was 4.5 kGy, AA neutralization was 80%, and m(AA):m(Am) was 2. The addition of MBA was 1.2% (m/V) [3,15]. The impact of the radiation dose rates ranging from 0.5 to 3.0 kGy/h on absorption is presented in Figure 4b. Absorption for both Qd and Qs increased with a dose rate of up to 1.5 kGy/h in distilled water (860 g/g) and in the 0.9 wt.% NaCl solution (85 g/g); then, a decreasing trend was observed. The dose rate is crucial for radiation-induced graft copolymerization, potentially determining the grafted branch length. Elevated dose rates intensify radical formation in the monomer, RCS, and water reaction mixture, enhancing both homopolymer formation and grafting reaction conversion [19,25]. High dose rates increase monomer radical recombination, forming homopolymers and reducing monomer radicals for graft copolymer initiation [6,7]. If the dose rate is excessively high, Qd and Qs water absorption decreases. The optimal dose rate was therefore determined to be 1.5 kGy/h.

#### 2.2.3. Effects of Radiation Dose on the Water Absorbency of SAPG

As the monomer-to-RCS weight ratio was 8, the dose was 4.5 kGy, the dose rate was 2.0 kGy/h, AA neutralization was 80%, and m(AA):m(Am) was 2. The addition of MBA was 1.2% (m/V) [3]. The impact of the radiation dose ranging from 2 to 8 kGy on the absorption of SAPG is presented in Figure 4c. Water absorbency increased with the absorbed radiation dose up to 5 kGy in distilled water (880 g/g) and in the 0.9 wt.% NaCl solution (88 g/g). This could have been due to the enhanced formation of radicals on the cellulose backbone, resulting in a high degree of crosslinking [26]. The subsequent increase in the dose reduced absorbency, as the addition of more radicals reinforced the termination step via bimolecular collision, which certain results in reduced chain lengths, resulting in different crosslinking densities and compact network structures [27]. In addition, higher irradiation might increase the amount of radiolysis products as well, i.e., ${OH}^{\cdot }$ and $e_{aq}^{-}$, which facilitating homopolymerization rather than the grafting reaction [26].

#### 2.2.4. Effect of AA Neutralization on the Water Absorbency of SAPG

**Figure 5:**
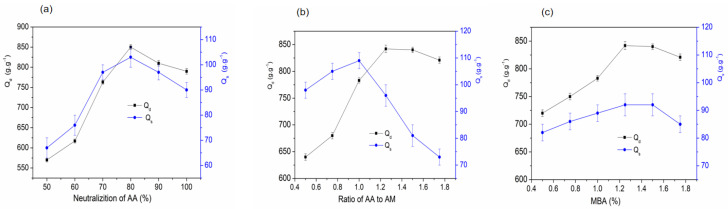
(**a**) Effect of AA-to-AM ratio on the water absorption rate. (**b**) Effect of neutralization degree of AA on the water absorption rate. (**c**) Effect of MBA on the water absorption rate.

As the monomer-to-RCS weight ratio was 8, the dose was 4.5 kGy, the dose rate was 2.0 kGy/h, and the m(AA):m(Am) was 2, The addition of MBA was 1.2% (m/V) [28]. The impact of the AA neutralization degree on SAPG water absorption is shown in Figure 5a. Absorption increased with AA neutralization degrees of up to 80% and then decreased with further neutralization to 100%. Optimal absorption (850 g/g) in distilled water and 85 g/g in the 0.9 wt.% NaCl solution occurred at 80% AA neutralization. Due to the cellulose’s intra-molecular and inter-molecular hydrogen bonding, the hydroxyls on β-glucose residues in compact-coil RCS cellulose molecules are more stable than those in small molecules [8]. As previously reported, hydroxyls on cellulose molecules can be replaced by OH- to form alkali cellulose in NaOH aqueous solutions [13]. This allows RCS cellulose to dissolve in NaOH aqueous solutions as alkoxide forms at low temperatures. After RCS dissolves in NaOH/urea aqueous solutions, adding AA gradually can neutralize most of the NaOH, resulting in a decrease in pH. The solution becomes slightly cloudy, but no precipitate forms. Urea is known to prevent cellulose aggregation through hydrophobic functions and the hydrophobic portion of cellulose. Thus, urea likely promotes stability and maintains a homogeneous aqueous cellulose solution. As the AA concentration increases, the proportion of -COO^−^ and Na^+^ ionic groups also increases until NaOH is fully neutralized, with 20% AA remaining non-neutralized. This generates osmotic pressure and electrostatic repulsion differences within the three-dimensional network structure, ultimately resulting in water absorption [13,26].

#### 2.2.5. Effect of AA-to-Am Ratio on the Water Absorbency of SAPG

As the monomer-to-RCS weight ratio was 8, the dose was 4.5 kGy, the dose rate was 2.0 kGy/h, and AA neutralization was 80%. The addition of MBA was 1.2% (m/V) [15]. The influence of the AA-to-Am weight ratio on SAPG water absorption is shown in Figure 5b. Water absorption increased with an increase in the AA-to-Am ratio until maximum absorption was achieved—849 g/g in distilled water and 84 g/g in the 0.9 wt% NaCl solution—at an optimal AA-to-AM ratio of 1.2. However, in the 0.9 wt.% NaCl solution, absorption increased from an AA-to-Am ratio of 0.5 to 1.00, and it decreased with further increases. COO^−^ in AA can form ionic bonds with water molecules, while CO-NH_2_ in Am can form hydrogen bonds [29]. Adjusting the AA-to-AM weight ratio modifies the proportion of the two hydrophilic groups (-CONH_2_ and -COONa), increasing polymer chain charges. This establishes electrostatic repulsion, expanding the chain network and increasing stiffness [30]. Increased -COO^−^ and Na^+^ groups boost osmotic pressure differences between the network’s interior and exterior [31]. Ions in saline water offset osmotic pressure, resulting in equilibrium ionization conversion and reduced water absorption in saline water if the AA-to-Am ratio exceeds 1. Additionally, reduced water absorption in SAPG with an AA-to-Am ratio over 1.25 could also result from weaker hydrogen bonding interactions among the COOH and amide groups.

#### 2.2.6. Effect of MBA on the Water Absorbency of SAPG

As the monomer-to-RCS weight ratio was 8, the dose was 4.5 kGy, the dose rate was 2.0 kGy/h, AA neutralization was 80%, and m(AA):m(Am) was 2 [20]. The addition of crosslinking agents (N,N’-methylene-bisacrylamide, MBA) enabled the polymer to form a complete network structure, which strengthened the resin’s physical retention ability relative to water molecules during water absorption. As shown in Figure 5c, with the increasing amount of MBA, the water absorption ratio of the product first increased, reached the highest value at 1.2% of the MBA (m/V), and then gradually decreased. When the amount of MBA was less than 1.2%, the water absorption ratio was relatively low because, with an insufficient amount of MBA, the crosslinking density was low, and the grafted copolymer hardened rapidly after water absorption. If the amount of crosslinking agent was too low, the network’ structure was not complete enough, resulting in a lower water absorption ratio [32]. As the amount of MBA increased, the space between the branched networks became too large, which hindered the swelling of the resin during water absorption, thus reducing the water absorption ratio. From the above, it could be concluded that the optimal amount of MBA was 1.2%.

### 2.3. Optimization of the Reaction Parameters

The orthogonal experiment was planned based on a four-factor, three-level design (SPSS v. 19.0, IBM Inc., Armonk, NY, USA), as presented in Table 1. Factor A denoted the ratio of the monomer-to-corn straw mass, represented as m_m_:m_CS_ (g/g). Factor B denoted the neutralization extent of AA. Factor C represented the radiation dose, denoted as dose (kGy). Factor D indicated the ratio of AA to Am, expressed as m_AA_:m_Am_ (g/g). For the A_2_B_3_C_3_D_2_ configuration, absorbency reached 98 g/g in the 0.9 wt% NaCl solution. The highest absorbency in the NaCl solution was realized with the A_2_B_3_C_2_D_2_ setup, and a distilled water absorbency of 1033 g/g was obtained. The effects of distilled water absorbency could be arranged as B > D > C > A (Table 1), while for the 0.9 wt% NaCl solution, the order was B > C > D > A (Table 1). Consequently, the optimal combination was determined to be A_2_B_3_C_2_D_3_ for distilled water and A_2_B_3_C_3_D_2_ for the 0.9 wt% NaCl solution. Under these parameters, CS-g-AA-co-Am was synthesized without heating or nitrogen protection, yielding an absorbency of 1033 g/g for distilled water and 98 g/g for the NaCl solution.

## 3. Conclusions

Corn straw (CS) was initially decrystallized in a NaOH/urea solution at low temperatures, followed by the preparation of a CS composite superabsorbent via graft copolymerization using RCS, Am, and AA under homogeneous conditions, initiated by gamma rays without heating. The effects of the RCS-to-monomer ratio, radiation dose, dose rate, and AA neutralization degree were explored. The resulting SAPG exhibited 1033 g/g water absorption in distilled water and 90 g/g in the 0.9 wt.% NaCl solution, with a radiation dose of 5 kGy, a dose rate of 1.5 kGy/h, an AA-to-Am mass ratio of 1.2, a monomer-to-RCS mass ratio of 7, and 90% AA neutralization. FTIR spectra confirmed the decrystallized CS and RCS composite SAPG formations, showing the characteristic casing bands of both CS and monomer units. XRD confirmed the crystallinity of CS, decrystallized RCS, and RCS composite SAPG, verifying grafting via radiation under homogeneous conditions. TG indicated lower thermal stability for RCS compared to SAPG. SAPG production introduced challenges, such as pollution, high energy consumption, and poor water retention in saline solutions. 

This is vital for SAPG use in agriculture, as SAPGs form a transparent gel without residual RCS particles [33]. Additionally, NaOH and urea act not only as solvents but also as reaction reagents, with urea serving as a fertilizer in agriculture [34]. The products exist as a resin without excess reagent emissions, aiding in reducing environmental pollution and simplifying the synthesis process due to the absence of heating preservation [35]. The results suggest a convenient SAPG preparation method with significant industrial application potential.

## 4. Materials and Methods

### 4.1. Materials

Acrylic acid (AA, analytical grade, chemically pure), NaOH, urea, acrylamide (Am, chemically pure), and N,N′-methylene-bisacrylamide (MBA, chemically pure) were sourced from Shanghai Chemical Reagent Corporation (Shanghai, China). The corn straw (CS) for this research was procured from the outskirts of Anhui, Hefei (China). Additional commercial solvents and reagents employed were of analytical grade and utilized without additional purification. Normal saline was made by dissolving 0.90 g NaCl in 99.10 mL of distilled water.

### 4.2. Pre-Treatment of CS

The CS was segmented and subjected to drying at 80 °C for a duration of 8 h. Following this, the dehydrated CS was processed through a milling machine and then sieved to achieve a particle size of 100 mesh. The milled CS dust was then weighed and suspended in pre-chilled NaOH/urea, where the concentration of NaOH was 6% (*w*/*w*) and the concentration of urea was 4% (*w*/*w*), in an aqueous solution at −12 °C, followed by a 15 h stirring process to yield a consistently clear solution. It was caused to precipitate by introducing anhydrous ethanol, and the resultant aggregated sediment was isolated via filtration. This sediment was subsequently cleansed with 95% ethanol and dried at 60 °C for a period of 48 h. Ultimately, the reconstituted corn straw was granulated and designated as RCS [36].

### 4.3. Creation of SAPG

A measured quantity of AA and MBA (the addition amount was the unit of grams per 100 mL of the volume of the system, m/V) was incorporated into the homogenous RCS and NaOH aqueous blend until a certain degree of neutrality was achieved, all while avoiding nitrogen protection. Subsequently, a calculated amount of AM was introduced to the combined solution and agitated for half an hour at ambient temperature (monomer: the mixture of AA and AM). The solutions were exposed to specific radiation dosages from ^60^Co gamma rays, emanating from a source located at the State Forestry Radiation Center in Hefei, China. Graft copolymerization occurred at room temperature. The moisture content of the product was approximately 50–70%. Post polymerization, the resultant hydrogel was divided into smaller chunks and left to dry for a night at 60 °C within a drying apparatus. These chunks were minced and sifted to a particle size of 200 mesh before being soaked in acetone for 5 h, over two times, to eliminate ungrafted monomers and additional substances. After filtering, the acetone residues were allowed to evaporate, followed by drying the substance at 60 °C until reaching a stable weight, resulting in pure SAPG powder [29].

### 4.4. Assessment of Water Absorption

The water absorption ability was evaluated at normal room temperature. A half-gram sample was submerged in distilled water and saline solution separately, enabling it to expand. Excess moisture was then eliminated with a 120-mesh sieve. The mass of the swollen samples was recorded until no further water dripped from them. The water absorption rate (Q, g/g) for the SAPG was computed using the following formula [4]:

$$Q=\frac{m_{2}-m_{1}}{m_{1}}$$

with *m*_1_ representing the dry sample mass and *m*_2_ the mass of the swollen sample (g).

### 4.5. Sample Analysis

The infrared (IR) spectra of the samples were captured through KBr pellets utilizing an FTIR spectrometer (Thermo Nicolet, Waltham, MA, USA). We weighed the dried sample and combined it with potassium bromide in the grinding dish at 0.5~1%. The mass ratio was uniformly mixed, and, then, at 44 wave resolution, 32 scans were conducted, followed by KBr tablet preparation.

The thermal resilience of the samples was documented with an HTG-1 thermogravimetric analyzer (Beijing Scientific Instrument Factory, Beijing, China), spanning a temperature range from 25 to 600 °C at a heating rate of 10 °C/min under a dry nitrogen flow of 40 mL/min.

Powder X-ray diffraction (XRD) assessments were conducted on an X-ray diffractometer, model DX 2700 (Haoyuan Ltd., Dandong, Liaoning, China), at 20 °C. The instrumental parameters included 40 kV and 40 mA, with Cu Ka radiation at λ = 0.15406 nm. The diffraction angle 2θ was scanned from 5° to 50° with 2° min^−1^ and a step increment of 0.02°.

For SEM, a Phenom PRO scanning electron microscope (Thermo Fisher Scientific, Eindhoven, The Netherlands) was used, at an acceleration voltage of 5 kV and 10 kV. The detector was a backscatter electron detector, with magnification of 160–135,000. Using double-sided tape on the sample stage of the scanning electron microscope, we took a small amount of dry powdered copolymer sample, placed it on the double-sided tape, and then used a bulb pipette to blow it apart to ensure even distribution. After that, we placed the sample in the sputter-coating device for carbon coating and gold plating. The electron gun acceleration was 5.0 kV, and the magnification was 1400×.

## Figures and Tables

**Table 1:** Orthogonal experiment results.

	A m_m_:m_SD_ (g/g)	B Neutralization of AA (%)	C Dose (kGy)	D m_AA_:m_Am_ (g/g)	Q_d_ in the Distilled Water (g/g)	Q_s_ in the Saline Solution (g/g)
1	1 (6:1)	1 (70)	1 (4)	1 (0.8)	701 ± 13	42 ± 3
2	1	2 (80)	3 (6)	2 (1.0)	829 ± 10	81 ± 4
3	1	3 (90)	2 (5)	3 (1.2)	977 ± 8	78 ± 2
4	2 (7:1)	1	3	3	869 ± 6	62 ± 2
5	2	2	2	1	752 ± 13	82 ± 4
6	2	3	1	2	986 ± 19	71 ± 3
7	3 (8:1)	1	2	2	934 ± 10	68 ± 2
8	3	2	1	3	814 ± 6	65 ± 6
9	3	3	3	1	736 ± 8	61 ± 2
$k_{1}$ ^*a*^	834 ± 10	835 ± 10	834 ± 13	730 ± 11		
$k_{2}$	869 ± 13	798 ± 10	888 ± 10	916 ± 11		
$k_{3}$	828 ± 8	900 ± 12	811 ± 8	887 ± 7		
*R* ^*b*^	41	112	77	186		
$k_{1}^{\prime }$ ^*c*^	67 ± 3	57 ± 2	59 ± 5	62 ± 3		
$k_{2}^{\prime }$	72 ± 3	76 ± 5	76 ± 3	73 ± 3		
$k_{3}^{\prime }$	65 ± 3	70 ± 2	68 ± 3	68 ± 3		
$R^{\prime }$ ^*d*^	7	19	17	11		
Q	A_2_	B_3_	C_2_	D_2_		

^*a*^ $k_{1}$ = (Σ the water absorbency in the distilled water of single-factor)/3. ^*b*^ $R_{1}=\max k_{1}-\min k_{1}$. ^*c*^ $k_{1}^{\prime }$ = (Σ the water absorbency in saline solution of single-factor)/3. ^*d*^ $R_{1}=\max k_{1}^{\prime }-\min k_{1}^{\prime }$. Q = the optimal conditions.

## Data Availability

Experimental data can be obtained by emailing the corresponding authors.
